# Cost analysis of tuberculin skin test and the QuantiFERON-TB Gold In-tube test for tuberculosis screening in a correctional setting in Dallas, Texas, USA

**DOI:** 10.1186/s12879-016-1901-8

**Published:** 2016-10-12

**Authors:** Ank E. Nijhawan, Princess A. Iroh, Larry S. Brown, Daniel Winetsky, Esmaeil Porsa

**Affiliations:** 1 Department of Internal Medicine, University of Texas Southwestern Medical Center, Dallas, Texas USA; 2 Parkland Health & Hospital System, Dallas, Texas USA; 3 Department of Health Systems Research, Parkland Health & Hospital System, Dallas, Texas USA; 4 Rutgers University Correctional Health Care, Rutgers University, Trenton, NJ USA; 5 Office of Strategy and Integration, Parkland Health and Hospital System, Dallas, Texas USA

**Keywords:** Tuberculosis, Jail, Screening, IGRA, TST, QFT-GIT

## Abstract

**Background:**

Tuberculosis (TB) disproportionately affects immigrants, HIV-infected individuals, and those living in crowded settings such as homeless shelters and correctional facilities. Although the majority of jails and prisons use a tuberculin skin test (TST) for latent tuberculosis infection (LTBI) screening, limited data exist on the clinical performance and costs of the TST compared to interferon gamma release assays (IGRAs) in this setting.

**Methods:**

A prospective pilot study comparing cost between TST and an IGRA (QuantiFERON Gold In-tube, QFT-GIT) for the detection of LTBI in a convenience sample of inmates entering the Dallas County Jail (DCJ) was conducted June–October 2014. Participants completed a risk questionnaire, TST placement, QFT-GIT testing, and were offered opt-out HIV-Ab testing. LTBI prevalence based on TST and QFT-GIT results, an evaluation of discordant results and a cost analysis are presented.

**Results:**

A total of 529 subjects were enrolled. The majority were male (75 %), and 46 % were Black, 29 % White, and 24 % Hispanic. Most (85 %) had been previously incarcerated. Over 28 % of participants were released prior to TST reading, with paired QFT-GIT and TST results available for 351 subjects. Of these, nine (2.6 %) tested positive by TST and 47 (13.4 %) tested positive by QFT-GIT. It costs $23.27 more per inmate per year to screen with QFT-GIT than TST in this population, though the cost per LTBI case detected was nearly three times higher for TST than QFT-GIT ($1247 v $460).

**Conclusions:**

We found a substantially higher rate of QFT-GIT positivity compared to TST in this sample of individuals entering the Dallas County Jail. Although no gold standard exists, this finding may indicate under-recognized LTBI in this setting. QFT-GIT as an initial screening tool was more time-efficient, had four-fold fewer labor costs and provided results on more individuals when compared with the TST. The overall cost of QFT-GIT was $23.27 more per inmate per year, though the cost per LTBI case detected was nearly three times higher for TST than QFT-GIT. Further research is needed to determine the long-term performance of IGRA testing in the correctional setting and the public health implications of pairing QFT-GIT screening with other tests for communicable diseases.

**Electronic supplementary material:**

The online version of this article (doi:10.1186/s12879-016-1901-8) contains supplementary material, which is available to authorized users.

## Background

Tuberculosis (TB) incidence in the US is declining; however certain vulnerable populations are disproportionately affected by TB, including immigrants, HIV-infected individuals, and those living in crowded settings such as homeless shelters and correctional facilities. In 2013, a total of 9582 cases of active TB disease were reported in the US and four states (California, Texas, New York, and Florida) accounted for over half of these cases [1]. The Dallas metro area has the fifth-highest incidence of TB among US cities [1]. Of the 1145 TB disease cases in Texas in this year; 116 (10.1 %) were diagnosed in a correctional facility. Given the overrepresentation of TB cases in the criminal justice system, Texas jails and prisons are required by law to screen all detainees for tuberculosis upon arrival. Many jail inmates have short incarcerations and return directly to the community after release, emphasizing the public health function and investment of local jails. In the Dallas County Jail 4300-5900 tuberculin skin tests (TSTs) are placed per month, requiring eight dedicated full time-equivalent nurses or medical aides, which includes staff and security time to visit inmates in their cells to read the TST result (personal communication, Dr. Esmaeil Porsa, 4/27/15). Nationally, an increasing proportion of the correctional budget is spent on screening and treating inmates for communicable diseases such as TB [2], the majority of whom rely on TST [3].

However, there are multiple limitations with using TST, an intradermal injection of purified protein derivative, for TB screening. Limitations include inter-operator variability in placement and reading, requirement of a second visit after 48–72 h for reading the TST (when 30 % of jail inmates have been released), cross-reactivity with Bacillus Calmette–Guérin (BCG) vaccine and nontuberculous mycobacteria, and variable interpretation of test results depending on patients’ prior testing and risk of infection. A more recent technology of interferon-gamma release assays (IGRAs) has been developed and involves testing a whole blood sample for a T cell response to tuberculosis-specific antigens. This test does not require a return visit, is not affected by BCG vaccination, has minimal impact from non-tuberculous mycobacteria and has fewer issues with inter-rater reliability. Several studies comparing TST with IGRAs in the incarcerated setting have found discordant test results, especially in individuals who had previously received BCG vaccine or who are foreign-born [4, 5]. No gold standard exists for the detection of latent tuberculosis infection (LTBI), though test performance can be compared by monitoring patients for the development of active tuberculosis disease. The specificity of IGRA testing is 98–100 % [6] versus 88.7 % for TST, with a negative predictive value (NPV) for progression to tuberculosis disease within 2 years of 99.8 % for QuantiFERON Gold In-tube (QFT-GIT, Qiagen) and 99.4 % for TST [7], according to a meta-analysis incorporating low, intermediate and high risk countries. Positive predictive value for developing tuberculosis disease in individuals who were close contacts or recent immigrants and tested positive for LTBI but refused prophylaxis was 2.8–14.3 % for QFT-GIT and 2.3–3.3 % for TST [6, 8]. The cost of IGRA testing varies, but is usually in the range of $35–40 compared to approximately $3–13 for TST [9, 10].

Although IGRA tests have been found to be cost-effective when compared to TST in settings where individuals require repeat testing such as in healthcare [10], limited data exists for this comparison in the incarcerated setting [11]. In this study, we sought to: (1) estimate the LTBI prevalence based on TST and an IGRA test (QFT-GIT) results in individuals entering a large county jail in Dallas, Texas and (2) measure the discordance of TST and QFT-GIT results in this setting in order to achieve our overarching aim: (3) to use prospective utilization data to compare costs between the TST and QFT-GIT test for LTBI screening. Our study emphasized the collection of primary data relevant to the specific demands and constraints of using these diagnostic modalities in correctional settings.

## Methods

### Study design

This study is a prospective pilot study comparing cost and clinical performance between the TST and the QFT-GIT test for the detection of LTBI in a convenience sample of inmates entering the Dallas County Jail (DCJ) between June and October 2014. The DCJ houses 6000–7000 inmates at any one time, with approximately 275 inmates entering and leaving the jail daily. The current protocol for LTBI screening is that all entering inmates have a TST placed, unless a result within 90 days is available or unless credible evidence indicates that they have previously tested positive for LTBI or TB disease, received prophylaxis for LTBI or received treatment for tuberculosis disease. For patients in these latter three categories, a Chest X-ray (CXR) is performed. The University of Texas Southwestern Medical Center Institutional Review Board (#042013-094) approved this study.

### Study population and recruitment

Adults entering the jail were approached for participation in the study in the classification area of the jail, where inmates await their housing assignment and where LTBI screening is performed. Eligible individuals had to be ≥ 18 years old, able to speak English, and able to provide informed consent. Of note, five individuals were not approached during the study period due to inability to speak English. Individuals with known prior positive TST or with a history of severe necrotic reaction to the purified protein derivative (PPD) used for TST were excluded from the study. All inmates provided written informed consent and authorization for use and disclosure of health information for research purposes.

### Study procedures

After informed consent was obtained, each subject completed a brief TB/HIV risk questionnaire assessing country of birth, BCG vaccination, past exposure to TB, history of homelessness, prior incarcerations, injection drug use and sexual history. Immediately following the questionnaire, blood samples were collected for HIV Ab testing (Immunochemiluminometric assay, with HIV-1/HIV-2 supplemental antibody confirmation, LabCorp, Inc.) and then for QFT-GIT testing (collected directly into QFT-GIT tubes) by the study phlebotomist. Of note, both study phlebotomists underwent training on how to draw and handle samples for QFT-GIT testing. In addition, HIV testing was offered to all study participants on an opt-out basis.

Following the blood draw, as part of routine care, participants completed the jail LTBI screening protocol, including assessment for symptoms (cough, hemoptysis, night sweats, unexplained weight loss) and placement of the TST by a trained jail health care worker. If it was discovered after enrollment that the participant had a prior positive TST (e.g. patient did not report this at time of enrollment, but this was discovered in the electronic medical record), this was recorded and a new TST was not placed. Participants who were discovered to have a prior positive TST after enrollment were not removed from the study. After 48–72 h, for inmates who had a TST placed and who remained incarcerated, induration at the site of injection was measured and reported in millimeters. An induration of ≥10 mm (or ≥5 mm in HIV-positive participants) was considered positive. Those with a positive TST underwent a CXR.

Blood specimens were sent on the day of collection via courier to Children’s Medical Center of Dallas Laboratory for QFT-GIT testing. Study staff were blinded to QFT-GIT results until the end of the study (in order to avoid bias in TST reading) unless a result was indeterminate or there was an insufficient sample, in which case the study team was notified and a repeat specimen was drawn if the participant was available and willing. The QFT-GIT was considered positive if ≥ 0.35 IU/ml [12]. HIV Ab tests were run per routine jail policy, which included notification of test results and risk reduction counseling.

By jail policy, any inmates who are identified as TST-positive undergo CXR screening. If the CXR is abnormal, the patient will undergo a work-up for TB disease. If CXR does not show any signs of active TB disease, the individual will be evaluated for initiation of treatment for LTBI. Current practice at the jail is to start high-risk individuals (HIV-infected, immunocompromised, chronically ill, recent conversions (within the past 2 years), or known contacts with a TB case) on LTBI treatment, others are referred to the health department after release. All QFT-GIT results, after being released at the end of the study, were managed with the same process as a positive TST.

### Sample size calculations and statistical analysis

For the cost comparison, sample size calculations were based on the assumption that each individual in the study would have both tests performed (paired testing) and included preliminary estimates of cost of testing, predicted staff time and positivity rates ($79.30 for TST and $77.90 for QFT-GIT). A sample size of at least 487 was chosen in order to detect a cost difference of $1.40 between the two testing modalities at 80 % power with a standard deviation of $11.

Baseline characteristics were tabulated by frequencies. We compared TST and QFT-GIT results using a 2X2 table for all individuals who completed both tests. The strength of this agreement was examined using Cohen’s kappa (κ) with a kappa statistic value of >0.75 representing excellent agreement beyond chance, 0.40 to 0.75 representing fair to good agreement beyond chance, and <0.40 representing poor agreement beyond chance. Statistical analyses were performed using SPSS (SPSS Inc. version 19, Chicago IL) and SAS (SAS Institute Inc. version 9.4, Cary, NC).

### Cost analysis

We performed a cost analysis taking the health program perspective, focusing on the cost components relevant to facility-based health care systems. In order to determine real-time human resource costs associated with LTBI screening, a time-in-motion analysis was performed for a subset of patients. A stopwatch was used to directly record the amount of healthcare and security staff time required for each step of the LTBI screening process, including TST placement, TST reading, QFT-GIT blood draw, time needed to perform and interpret a CXR (including security time, radiology technician time, radiologist time, physician interpretation time). The clinical and laboratory staff costs were calculated using DCJ and Parkland Health and Hospital System hourly salary tables for employees.

We included additional direct material costs such as retail prices for all supplies required for TST (including tuberculin, syringes, needles, etc.) and chest radiography as well as costs of testing and commercial kits for the QFT-GIT. Costs were estimated in 2013 US dollars. On-site capital costs for both TST and QFT-GIT (storage and refrigeration of testing materials as well as staff training) contributed negligibly to the total cost of each strategy and were similar across strategies. These were therefore excluded from the final analysis. Off-site indirect and capital costs for QFT-GIT were assumed to be incorporated into the retail price for commercial kits.

**Fig. 1:**
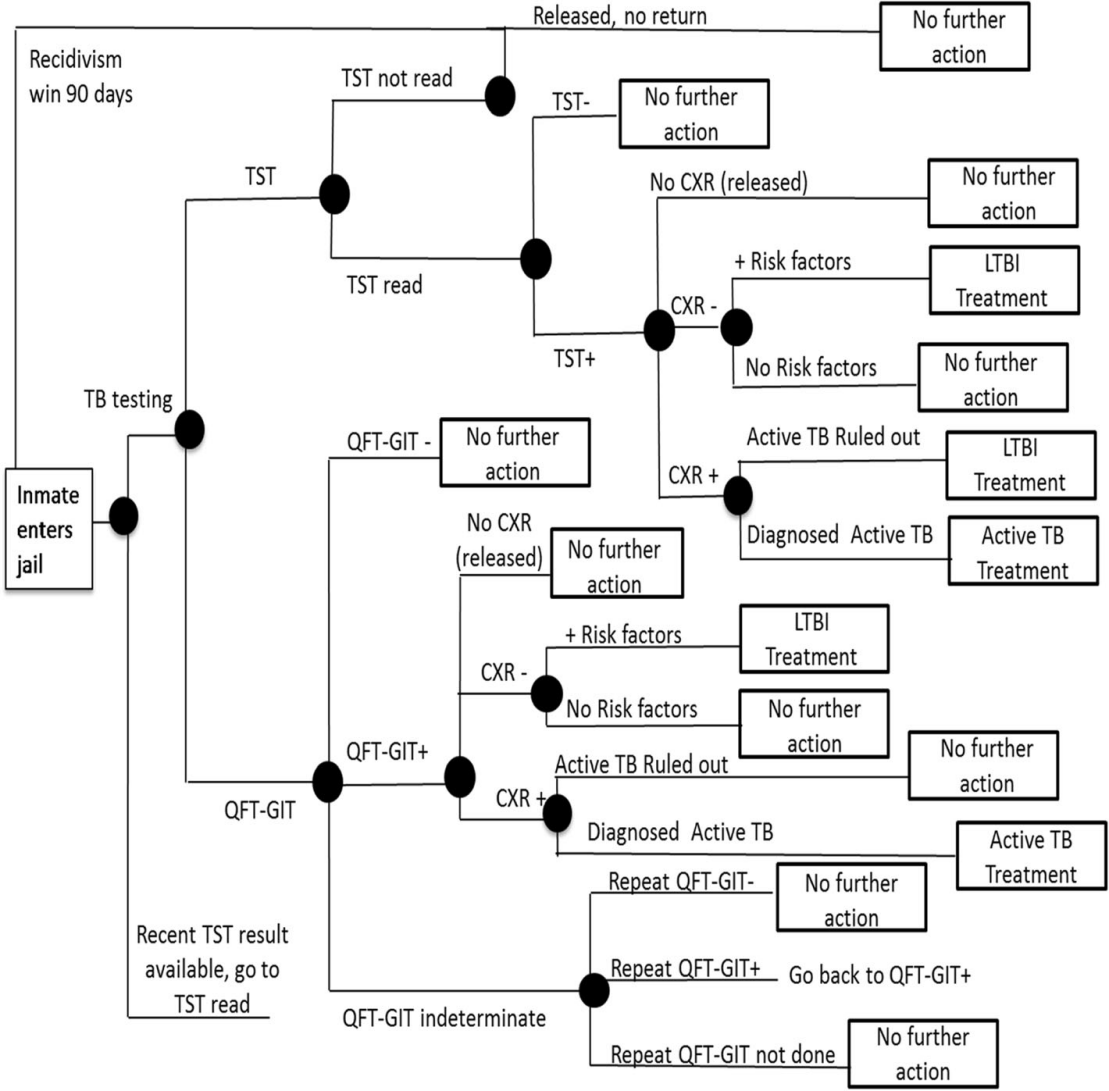
Decision tree: LTBI screening using TST and an IGRA (QFT-GIT) test method. A decision tree model for LTBI screening using two testing methods (TST and QFT-GIT) upon entry to Dallas County Jail. □ = decision node; ○ = event node; ⊲ = end of one event

A cost analysis was performed using a simple deterministic decision-tree model (Fig. 1) using Treeplan (Treeplan software, San Francisco, CA). The model uses static probabilities to project the costs and consequences of two alternative screening strategies over a 1 year period. Probabilities were calculated using the following primary study data: positivity rates of TST and QFT-GIT during the study, proportion with indeterminate QFT-GIT results and repeat testing results, proportion of study participants released prior to TST reading, proportion from whom a blood sample could not be obtained for QFT-GIT testing and proportion of participants who left prior to CXR completion. In addition, general jail release data from 2012 and 2013 were analyzed to determine 12 month recidivism rates for individuals released before and after 3 days of incarceration (time window assumed for TST to be read and QFT-GIT resulted). For each strategy projected in our model, one iteration of recidivism was considered, and screening results were considered valid for 12 months. For individuals released prior to completion of TST reading or follow up testing for indeterminate QFT-GIT, costs associated with “wasted” or incomplete tests were included into final estimates of cost per test completed and cost per LTBI case detected. Basic sensitivity analyses were performed to determine the effect of certain assumptions (TST positivity cutoff for QFT-GIT positivity, costs of labor in time-in-motion and unit price of QFT-GIT) on the cost difference between the two testing strategies. Lastly, a cost per LTBI case detected was calculated using the decision tree model to estimate the total annual cost for each testing strategy, divided by the number of LTBI cases (defined as positive test result and completion of CXR) detected by each strategy.

## Results

### Baseline characteristics

Of 576 subjects who were screened for participation in the study, 529 subjects (92 %) were enrolled. The majority were male (75 %), and 46 % were Black, 29 % White, and 24 % Hispanic. In addition, 17 % reported ever injecting drugs, 14 % had stayed in a homeless shelter and 85 % had been previously incarcerated. Only 1 % noted prior TB, 3 % reported an exposure to someone with tuberculosis and 1 % reported TB symptoms (Table 1).

**Table 1:** Baseline characteristics

Characteristic	Overall *529 (100 %)*
Gender	
Male	397 (75 %)
Age, mean (years)	33.5
18–29	242 (46 %)
30–39	137 (26 %)
40–49	91 (17 %)
> 50	59 (11 %)
Ethnicity	
Hispanic	128 (24 %)
Non-Hispanic	303 (57 %)
Unknown	97 (18 %)
Race	
Black	244 (46 %)
White	151 (29 %)
Native American^a^	21 (4 %)
Asian	4 (1 %)
Pacific Islander^b^	1 (<1 %)
Other	122 (23 %)
Non US born	19 (4 %)
TB/HIV Risk Factors	
Ever stayed in a homeless shelter	75 (14 %)
First incarceration	81 (15 %)
Ever Injected Drugs	89 (17 %)
Tested for HIV in past	376 (71 %)
MSM	20 (4 %)
HIV +	13 (2 %)
Past positive TB	6 (1 %)
Ever treated for TB	6 (1 %)
Vaccinated with BCG	63 (12 %)
Ever been exposed to TB	15 (3 %)
TB symptoms	
Cough 3 weeks	2 (<1 %)
Hemoptysis	1 (<1 %)
Night sweats	4 (1 %)
Unexplained weight loss	2 (<1 %)
Any of the above	7 (1 %)

*QFT-GIT* QuantiFERON-TB Gold In-Tube, *TST* tuberculin skin test, *US* United States of America, *MSM* men who have sex with men, *HIV* human immunodeficiency virus, *TB* tuberculosis, *BCG* bacille Calmette-Guérin, *CXR* chest x-ray)

^a^Refers to American Indian and Alaskan Natives

^b^Refers to Pacific Islander and Native Hawaiian

**Fig. 2:**
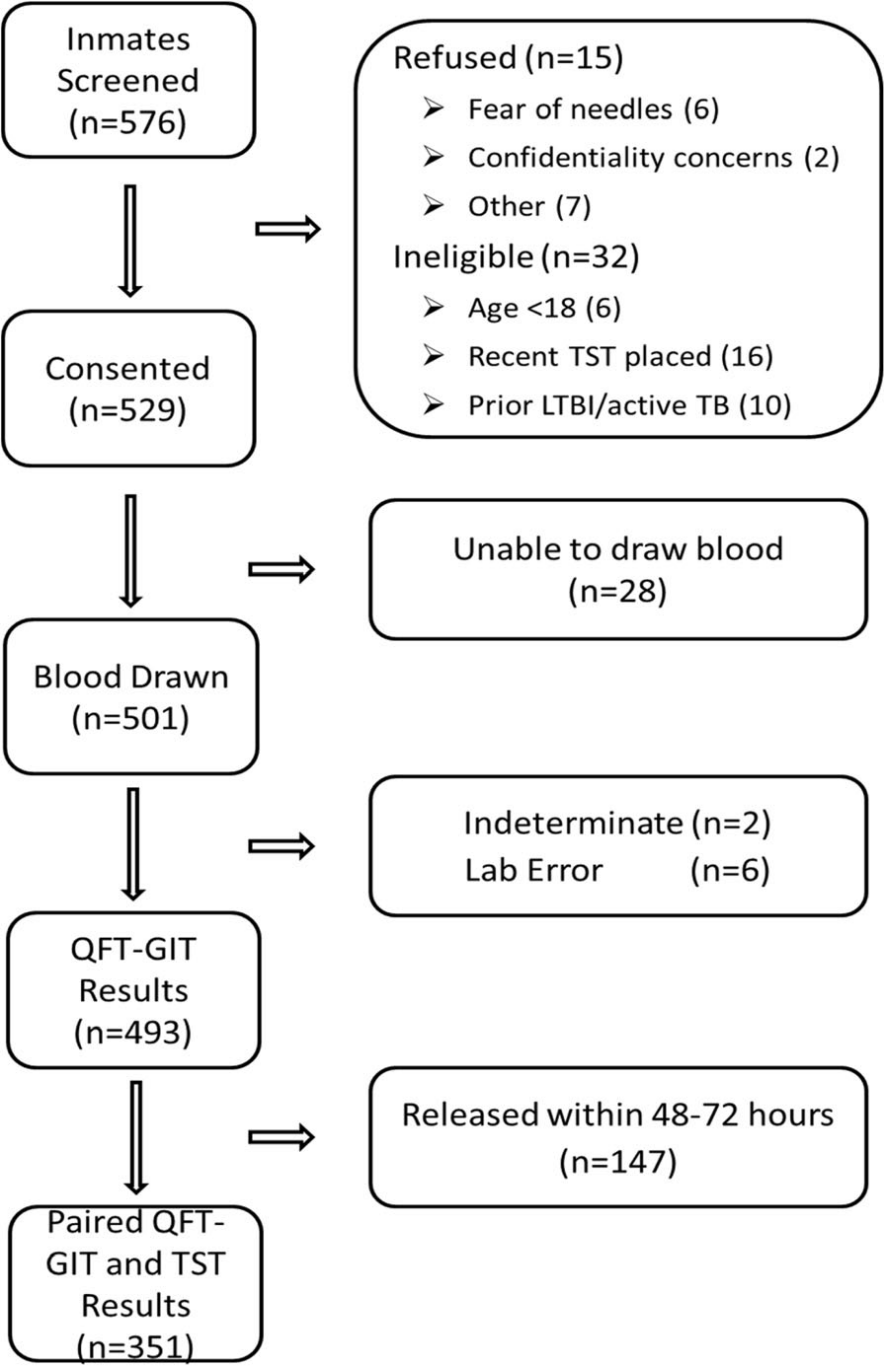
Flow diagram: Study enrollment and availability of paired results

Of all enrolled subjects, 520 (98 %) had a TST placed, and a blood sample was successfully collected from 501 (95 %) for QFT-GIT testing. Over a quarter of enrolled subjects, 147 (28 %), were released from DCJ prior to having their TST read. Of the QFT-GIT tests, two (0.4 %) had indeterminate results and six had incomplete results due to lab error (Fig. 2). Paired results of screening for LTBI by QFT-GIT and TST were available for 351 subjects. Of these, 9/351 (2.6 %) tested positive by TST and 47/351 (13.4 %) tested positive by QFT-GIT, of whom five had concordant positive TST and QFT-GIT results, with a kappa statistic of 0.14 (Table 2). Of all 493 QFT-GIT tests with an interpretable result, 66 (13.4 %) were positive. Of these, 30/66 (45 %) had quantitative values ≥1 IU/ml.

**Table 2:** Paired Results of testing with QFT and TST

	QFT-GIT positive	QFT-GIT negative	Total
TST positive	5	4	9
TST negative	42	300	342
Total	47	304	351

TST positivity rate: 9/351 = 2.6 %

QFT-GIT positivity rate: 47/351 = 13.4 %

Kappa score: 0.142

*QFT-GIT* QuantiFERON-TB Gold In-Tube, *TST* tuberculin skin test

Bivariate analyses found that male gender, having stayed in a homeless shelter, having ever injected drugs, prior treatment for TB and having a TST ≥5 mm were all associated with a positive QFT-GIT (Table 3).

**Table 3:** Comparison of frequency of patient characterisitcs between QFT-GIT positive vs. QFT-GIT negative

Variable	QFT-GIT positive (*N* = 66)	QFT-GIT negative (*N* = 427)	*P* value
Male gender	58 (87.9)	324 (75.9)	0.03
Age (Median (25^th^, 75^th^))	33 (23, 44)	31 (24, 42)	0.52
Age, by category			
18–29	28 (42.4)	201 (47.1)	0.54
30–39	14 (21.2)	108 (25.3)	
40–49	15 (22.7)	72 (16.9)	
> 50	9 (13.6)	46 (10.8)	
Hispanic ethnicity	21 (39.6)	97 (27.6)	0.07
Race			
Black	26 (39.4)	201 (47.1)	0.46
White	20 (30.3)	121 (28.3)	
Other	20 (30.3)	105 (24.6)	
Non US born	2 (3.1)	16 (3.8)	1.00
Stayed in homeless shelter	14 (21.2)	57 (13.4)	0.10
>1 incarceration	58 (89.2)	358 (84.2)	0.30
Ever Injected Drugs	16 (24.6)	60 (14.2)	0.03
HIV +	1 (1.5)	12 (2.8)	1.00
Tested for HIV in past	45 (68.2)	307 (71.9)	0.62
Ever treated for TB	3 (5.0)	2 (0.5)	0.02
Vaccinated with BCG	8 (14.3)	50 (14.1)	0.97
Ever been exposed to TB	4 (6.5)	11 (2.8)	0.14
TST read	47 (73.4)	304 (71.2)	0.71
TST > 5 mm	4 (8.5)	4 (1.3)	0.01
TST > 10 mm	2 (4.3)	3 (1.0)	0.14
TB symptoms			
Cough 3 weeks	1 (1.6)	1 (0.2)	0.24
Hemoptysis	0 (0.0)	1 (0.2)	1.00
Night sweats	1 (1.6)	3 (0.7)	0.43
Unexplained weight loss	0 (0.0)	2 (0.5)	1.00
Any of the above	1 (1.5)	6 (1.4)	1.00

*US* United States of America, *HIV* human immunodeficiency virus, *TB* tuberculosis, *BCG* bacille Calmette-Guérin, *TST* tuberculin skin test

Of 529 subjects offered opt-out HIV testing, 471 (89 %) tested negative, 13 (2.5 %) were confirmed as known HIV-infected individuals and one (0.2 %) had a preliminary positive result. In addition, 39 subjects did not have a result due to blood draw failure (26, 4.9 %), insufficient quantity of blood (8, 1.5 %), missing result (5, 0.9 %) or refused HIV testing (5, 0.9 %).

### Time-in-motion analysis

On average, TST placement and interpretation required 15 extra minutes of TB nursing/technician time and three extra minutes of security time compared to QFT-GIT testing, making the labor cost of screening nearly four times more for TST than QFT-GIT (Table 4). The total cost for completion of a single CXR, based on stopwatch times for each step, was $31.

**Table 4:** Time-in-Motion Results for TB Screening and Chest X-ray Completion

Procedure	Number of Measurements	TST staff time	TST staff cost	QFT-GIT staff time	QFT-GIT staff cost
*TB Screening*					
TST placement	15				
TB Nurse/Technician time		4 min (2–6 min)	$1.80	--	--
Security time		3 min (1–5 min)	$1.38	--	--
TST reading – 3 towers	64				
TB Nurse/Technician time		6 min (2–8 min)	$2.70	--	--
Security time		3 min (0.5–7 min)	$1.38	--	--
TST result entry	27				
TB Nurse/Technician time		8 min (3–12 min)	$3.60	--	--
QFT-GIT blood draw	23				
TB Nurse/Technician time		--	--	3 min (1–5 min)	$1.35
Security time		--	--	3 min (1–5 min)	$1.38
Total TB Nurse/Technician screening time		18 min	$8.10	3 min	$1.35
Total Security screening time		6 min	$2.76	3 min	$1.38
**Total TB screening staff cost**			**$10.86**		**$2.73**
*Chest X-ray*					
X-ray Technician time	13	5 min (3.5–7 min)	$2.83	5 min (3.5–7 min)	$2.83
Security time	13	26 min (10–50 min)	$11.98	26 min (10–50 min)	$11.98
Onsite MD interpretation time	13	2 min (0.5–5 min)	$3.30	2 min (0.5–5 min)	$3.30
Formal radiology reading		--	$13.00	--	$13.00
**Total Chest X-ray completion cost**			**$31.11**		**$31.11**

Bold text: Total costs for TB screening labor, Chest X-ray completion and LTBI treatment

*TB* tuberculosis, *QFT-GIT* QuantiFERON-TB Gold In-Tube, *TST* tuberculin skin test

### Cost analysis

Time-in-motion data were combined with local DCJ data to perform a cost analysis. Cost components were assessed for each strategy to determine total annual cost, cost per test completed and cost per LTBI case identified (Table 5). Probability and cost inputs are listed in Additional file 1: Table S1. It was assumed that CXRs would be completed for all non-released individuals with a positive QFT-GIT (since QFT-GIT results were blinded until end of study). Cost per test for LTBI screening using TST was $18.70 and using QFT-GIT was $41.97 or $23.27 more per inmate when screening with QFT-GIT versus TST (Additional file 1: Table S1, final cost analysis tree). The total cost per LTBI case detected was $1246.60 for TST and $459.87 for QFT-GIT (Table 5).

**Table 5:** Cost per LTBI Case Detected based on Total Annual Cost Estimates for TST and QFT-GIT Testing Strategies

	Direct labor cost per test (health care)	Direct labor cost per test (custody)	Material cost per test	Total annual cost to facility	Total number of tests completed	Cost per per test completed	Cost per LTBI case detected
TST	$8.10	$2.76	$8	$1,010,995.77	38928	$25.97	$1246.60
QFT-GIT	$1.35	$1.38	$37^a^	$2,272,386.29	50542	$44.96	$459.87

Indirect labor costs, overhead and on-site capital costs comprised negligible contributions to total cost and were assumed to be similar between strategies so were not included. ^a^Off-site capital costs for QFT-GIT were assumed to be included in the market price for laboratory services, which is listed here as the estimated material cost. Tests were considered completed if the results were obtained and returned to the patient. Detection of an LTBI case required a positive test and a completed chest radiograph with no evidence of active TB disease

### Sensitivity analyses

We tested several assumptions which could impact the cost difference between the two tests. For example, if the cutoff for QFT-GIT were higher (e.g. >1 IU/ml), then the cost difference per test would be $21.67 more for QFT-GIT than TST; if we also assume that TST positivity were at average DCJ rates (5.7 %), the cost difference declines to $21.14. Changes in estimates for the medical staff and security costs of reading TSTs diminish the cost difference more substantially to $17.60 if we assume labor costs that are twice as high as originally estimated. The cost difference was most sensitive to changes in the cost of the QFT-GIT test itself. If the QFT-GIT unit cost were $25, the overall cost difference would drop to $11.79 more per inmate for QFT-GIT than TST. If including higher estimates for staff/security costs and $25 price of QFT-GIT the cost difference is $5.69 more for the QFT-GIT per inmate.

### Validation of study results

Due to the relatively low rate of TST positivity during the study (prior data would have predicted a positivity rate of 5.7 % rather than 2.6 %), an internal audit was performed to assess TST reading. After TST reading had been completed per routine by jail health staff, an auditor repeated the TST reading on the same day for 73 inmates, of whom seven (9.5 %) whose original results were reported as negative were determined to have a positive result.

Due to relatively high QFT-GIT results, samples were sent to an outside reference lab (LabCorp, Inc.) as well as the study lab (Children’s Medical Center). Samples were sent from 10 individuals (blood sent to both labs on the same day) and identical results were obtained for all 10 QFT-GIT tests which were performed. Of note, one individual who had previously had a positive QFT-GIT test now had a negative test from both labs.

## Discussion

In this study comparing TST with QFT-GIT testing for latent tuberculosis in a large urban county jail, we found an unexpectedly high positivity rate of QFT-GIT, 13.4 %, compared to a unexpectedly low TST positivity rate of 2.6 %. We also found that performing a blood draw for TB testing was acceptable (92 % agreed to testing), was more time efficient (took 18 fewer minutes of overall time), had lower labor costs (4 fold less) and provided a test result for more inmates (493 v. 373). Our cost analysis, which incorporates test positivity as determined by the study, local DCJ data, and time-in-motion results, found that overall the QFT-GIT costs $ 23.27 more per test completed than the TST. However, the cost per LTBI case detected was 2.7 times more using the TST compared to the QFT-GIT method.

Although it is difficult to define the true prevalence of LTBI in this high-risk population due to the lack of a gold standard test, we have explored several explanations for the unexpected TST and QFT-GIT results. First, the lower than expected TST rate is not consistent with the general population LTBI prevalence of 4.2 % estimated by the National Health and Nutrition Examination Survey, 1999–2000, an estimate of US households. In this house-to-house survey, higher LTBI prevalence was associated with poverty, lower education and being foreign-born, African American or Mexican-American [13]. An international meta-analysis comparing the sensitivity of TST with QFT-GIT in active TB disease found a higher sensitivity of QFT-GIT (80 %) compared to TST (65 %) [14], indicating that the low TST positive rate could be due in part to false negative results. Another possibility is that some of the QFT-GIT tests represent false positive results, though prior reports have been limited to healthcare workers in low-risk settings [15, 16]. Reproducibility has been a concern with IGRAs, with some studies reporting high rates of reversion (from negative to positive or vice versa), potentially related to handling techniques (tube agitation, transport time to lab, incubation time) or the selection of a relatively low cut-off value which could produce varying results on the same individual over time, within a single laboratory or between different laboratories.[17, 18] Although only a small number of QFT-GIT results in our study were validated at an outside lab, 10/10 (100 %) had the same result in both labs and the overall rate of indeterminate results was low (1 %) which support reproducibility and indicates that the high positivity is unlikely to be due to handling or laboratory techniques.

Several epidemiologic factors support a high rate of LTBI in this population and may indicate that many of the QFT-GIT results represent true positives. First, other jails who use QFT-GIT for screening have also found high rates of positivity, as Rikers Island Jail reported a QFT-GIT positivity of 10 % when screening with this test was first implemented, though subsequent positivity rates have been near 5 % (personal communication, Ross MacDonald, MD, 11/12/14), with similar trends in the Ohio prison system using IGRA for LTBI screening (initial positivity 7 %, down to 3.7 % 6 months later) [19]. Second, Dallas experienced a tuberculosis outbreak in 2012, which was investigated by the Centers for Disease Control (CDC) and was centered in the homeless population (personal communication, Dr. Esmaeil Porsa, 11/17/2014). Although that investigation did not find any linked TB transmissions in the DCJ, given the high rate of incarceration among homeless individuals, the current high LTBI detection rate could reflect exposure to TB which occurred during a local outbreak.

Our study also found a relatively low rate of TST positivity. We specifically excluded those with a patient-reported prior positive TST since these individuals are typically excluded from routine IGRA testing. We also excluded those who did not speak English, thereby potentially excluding foreign-born individuals who had received BCG vaccine and are more likely to have a false positive TST (though foreign born also more likely to have true positive). In addition, TST reading error by TB staff, which highlights the subjectivity and variability in test interpretation with the skin test, also contributed to low positive TST rates.

Overall agreement between TST and QFT-GIT was quite low in this study, similar to other studies [4, 20, 21], and there may be multiple drivers of this discordance. It is possible that a higher cutoff value for positive QFT-GIT and more rigorous TST interpretation may have resulted in fewer discordant results. For the five individuals with both a positive TST and QFT-GIT, their quantitative QFT-TB Ag level results ranged from 1.46- >10 IU/ml. Those with negative TST and positive QFT-GIT had a mean quantitative QFT-TB Ag level of 1.57 IU/ml (median 0.65 IU/ml, range 0.4- > 10 IU/ml) suggesting most are true positives. Given the recognized shortcomings of the TST and the high specificity of QFT-GIT, it is possible that the TST missed 89 % (42/47) of LTBI infections.

With regards to direct program costs, the QFT-GIT carries a greater expense per test ($23.27 more per person) than TST but may be the preferred test in certain populations. In particular, IGRAs have better specificity in populations with high BCG vaccination rates [22]. In this study, many inmates who reported BCG vaccination were not foreign-born, and study participants seemed to confuse BCG vaccine and TST testing, therefore we do not feel that this self-reported variable is accurately reflected in our data. By only including English speakers and excluding those with a prior positive TST, our study likely underrepresents those with prior BCG vaccination entering the jail (thereby under-representing the value of QFT-GIT). Also, the study population had a relatively high prevalence of HIV infection (2.5 %), and although some studies favor the use of IGRAs in HIV patients [23, 24], there is no consensus on whether IGRAs are superior to TST and current Department of Health and Human Services guidelines for HIV opportunistic infection management suggest that either, but not both, can be used for LTBI screening in HIV patients [25]. According to CDC guidelines, the use of IGRAs is also indicated for groups with historically low rates of follow up for TST reading, such as homeless individuals and drug users [8]. As 28 % of our study population was released prior to TST reading, this may favor IGRA testing in this setting. In addition, if individuals also require LTBI screening for other settings (shelters, drug rehabilitation center), communication of LTBI screening results from the jail may result in shared cost savings. Lastly, although this study does not take into account the detection of active TB disease, QFT-GIT was more sensitive than TST in detecting active TB disease (80 v 65 %) per a meta-analysis [14].

Our economic evaluation focused specifically on the cost components most relevant in correctional settings, where human resource utilization is a central concern. As such, we aimed to perform a cost analysis based on real-time primary data from a large county jail. A full assessment of the effectiveness of TST- and IGRA-based screening strategies in preventing the subsequent development of active TB disease was outside the scope of this study, and would involve the projection of costs and health benefits accrued to individuals after release from the jail facility where our primary data was collected. However, in estimating the cost per case of LTBI detected, we were able to facilitate a basic evaluation of the relative cost-effectiveness of the alternative testing strategies being considered for settings of pre-trial detention, with regards to their most direct purpose––to identify individuals in whom preventive therapy is indicated.

Multiple cost comparison studies and cost effectiveness analyses of LTBI screening have been published previously. Generally, these have favored IGRA alone or TST followed by IGRA as the most cost favorable strategies, though studies vary by location, population and assumptions about test performance and cost [26, 27]. A recent analysis of the relative cost-effectiveness of IGRA-based screening strategies for different groups with high risk of TB found that their use was cost-effective compared to TST for foreign born individuals or those with HIV (based on an incremental cost effectiveness ratio of less than $100,000 per quality adjusted life year gained), but not for other vulnerable populations such as homeless individuals, drug users and former prisoners [9]. With regards to studies performed specifically in the incarcerated population, Schwartz et al. found high rates of discordance between TST and IGRA (TST+/IGRA-) testing among BCG vaccinated individuals in a Canadian prison, favoring IGRAs as a useful screening tool [5]. Kowada et al. found that QFT-GIT screening was more cost effective than TST in Japanese prisons, although a high rate of BCG vaccination (97 %) and treatment costs were assumed [28]. Lastly, there are also data to support miniature CXR (a photograph of an Xray fluoroscopic image historically used in mass TB screenings) for TB screening in the incarcerated setting, which was found to be more cost effective than TST ($9600 v $32100 per case identified) or symptom screening ($54,100 per case identified) in US jails [24]. Our study contributes to this literature by indicating that QFT-GIT was more efficient, provided more tests results and identified >5 times more cases of LTBI than TST.

This study has some important limitations. First, it is a single site study with a relatively small sample size. However, we feel that given the high rate of enrolment relative to those approached that this sample accurately reflects our local jail population. Second, we used local cost data and therefore these results may not be generalizable to other settings. However, since real-time cost data were used, including time-in-motion data, very few assumptions were made in our cost calculations. Some on-site indirect and capital costs (e.g storage, training) were not measured and assumed to be similar across strategies, but these contributed negligibly to the total cost of each strategy. Also, we conducted informal sensitivity analyses to test the impact of the assumptions that we did make. Third, we did not include the cost of LTBI treatment, nor did we project the cost of the averted consequences of detecting active tuberculosis disease, such as the costs of a contact investigation or the costs of treatment and its outcomes (potentially leading to an underestimate of the benefits of QFT-GIT) as this was beyond the scope of this study. Therefore, it is not possible to conclude from our analysis whether the increased program expenses we estimated for QFT-GIT are justified on the basis of its potential increased effectiveness. Lastly, this study was cross-sectional and spanned 4 months. Future investigation with longitudinal follow-up data may provide additional information about the predictive value of TST or QFT-GIT for the development of active tuberculosis disease in this setting.

## Conclusions

In summary, our study found a high positivity rate of QFT-GIT compared to TST in inmates entering a large urban county jail, though results may have been skewed by the exclusion of inmates with a prior positive TST. QFT-GIT testing in this setting was feasible and when compared to TST, it was more time efficient with more individual test results available. The QFT-GIT cost $23.27 more per inmate to perform than the TST, though depending on test positivity and labor costs, this additional cost may be considerably lower. The cost per LTBI case detected was 2.7 times lower for QFT-GIT than TST, and TST may have missed as much as 89 % of LTBI cases. There is a need for further cost-effectiveness data in jails and prisons to determine the true downstream costs of missing latent and active TB disease by TST. Obtaining a blood sample upon entry into jail provides an important public health opportunity, including testing for other communicable diseases, such as HIV, syphilis and hepatitis B and C. Other studies have found that pairing testing with QFT-GIT testing was feasible and useful in the incarcerated setting [29]. Decisions on how best to screen for TB in jails and prisons will depend on local prevalence of disease and site specific costs of screening and treatment. Cost sharing and clinical partnerships with community organizations and public health entities may increase the long-term public health impact of testing and treatment in the criminal justice system.

## Additional files

Additional file 1: Table S1. Decision tree probabilities and cost-analysis for LTBI screening at DCJ. This table includes all of the inputs utilized for the decision tree, including the probabilities and costs for each decision node. (DOCX 19 kb)
Additional file 2: Final Cost Analysis Tree. This file contains the structure of the cost analysis tree used for this study, which assumes that repeat testing for LTBI screening is performed annually for individuals who are incarcerated more than once. (XLSX 53 kb)

## Abbreviations

BCG: Bacillus Calmette–Guérin
CDC: Centers for disease control and prevention
CXR: Chest x-ray
DCJ: Dallas County Jail
HIV: Human immunodeficiency virus
IGRA: Interferon gamma release assay
LTBI: Latent tuberculosis infection
MSM: Men who have sex with men
QFT-GIT: QuantiFERON Gold In-tube
TB: Tuberculosis
TST: Tuberculin skin test
US: United States of America, Tech = technician.

## References

[1] CDC (2014). Reported Tuberculosis in the United States, 2013.

[2] Kinsella C (2004). Trends alert: corrections health care costs.

[3] Roberts CA, Lobato MN, Bazerman LB, Kling R, Reichard AA, Hammett TM (2006). Tuberculosis prevention and control in large jails: a challenge to tuberculosis elimination. Am J Prev Med.

[4] Porsa E, Cheng L, Seale MM, Delclos GL, Ma X, Reich R, Musser JM, Graviss EA (2006). Comparison of a new ESAT-6/CFP-10 peptide-based gamma interferon assay and a tuberculin skin test for tuberculosis screening in a moderate-risk population. Clin. Vaccine Immunol.

[5] Schwartz IS, Bach PJ, Roscoe B, Majury A, Hopman WM, Ellis E, Garrahan T, Smith J, Barkley R, Panaro L (2014). Interferon-gamma release assays piloted as a latent tuberculous infection screening tool in Canadian federal inmates. Int. J. Tuberc. Lung Dis.

[6] Diel R, Goletti D, Ferrara G, Bothamley G, Cirillo D, Kampmann B, Lange C, Losi M, Markova R, Migliori GB (2011). Interferon-gamma release assays for the diagnosis of latent Mycobacterium tuberculosis infection: a systematic review and meta-analysis. Eur. Respir. J.

[7] Diel R, Loddenkemper R, Nienhaus A (2012). Predictive value of interferon-gamma release assays and tuberculin skin testing for progression from latent TB infection to disease state: a meta-analysis. Chest.

[8] Mazurek GH, Jereb J, Vernon A, LoBue P, Goldberg S, Castro K (2010). Updated guidelines for using interferon gamma release assays to detect mycobacterium tuberculosis infection - United States, 2010. MMWR Recomm. Rep.

[9] Linas BP, Wong AY, Freedberg KA, Horsburgh CR (2011). Priorities for screening and treatment of latent tuberculosis infection in the United States. Am J Respir Crit Care Med.

[10] de Perio MA, Tsevat J, Roselle GA, Kralovic SM, Eckman MH (2009). Cost-effectiveness of interferon gamma release assays vs tuberculin skin tests in health care workers. Arch Intern Med.

[11] Kowada A (2013). Cost-effectiveness of interferon-gamma release assay for entry tuberculosis screening in prisons. Epidemiol Infect.

[12] QuantiFERON-TB Gold [Package insert] [http://usa.quantiferon.com/irm/content/PI/QFT/2PK/US.pdf]. Accessed 2 Dec 2015.

[13] Bennett DE, Courval JM, Onorato I, Agerton T, Gibson JD, Lambert L, McQuillan GM, Lewis B, Navin TR, Castro KG (2008). Prevalence of tuberculosis infection in the United States population: the national health and nutrition examination survey, 1999-2000. Am J Respir Crit Care Med.

[14] Sester M, Sotgiu G, Lange C, Giehl C, Girardi E, Migliori GB, Bossink A, Dheda K, Diel R, Dominguez J (2011). Interferon-gamma release assays for the diagnosis of active tuberculosis: a systematic review and meta-analysis. Eur. Respir. J.

[15] Dorman SE, Belknap R, Graviss EA, Reves R, Schluger N, Weinfurter P, Wang Y, Cronin W, Hirsch-Moverman Y, Teeter LD (2014). Interferon-gamma release assays and tuberculin skin testing for diagnosis of latent tuberculosis infection in healthcare workers in the United States. Am J Respir Crit Care Med.

[16] Fong KS, Tomford JW, Teixeira L, Fraser TG, van Duin D, Yen-Lieberman B, Gordon SM, Miranda C (2012). Challenges of interferon-gamma release assay conversions in serial testing of health-care workers in a TB control program. Chest.

[17] Gaur RL, Pai M, Banaei N (2013). Impact of blood volume, tube shaking, and incubation time on reproducibility of QuantiFERON-TB gold in-tube assay. J Clin Microbiol.

[18] Joshi M, Monson TP, Woods GL (2012). Use of interferon-gamma release assays in a health care worker screening program: experience from a tertiary care centre in the United States. Can Respir J.

[19] Transitioning from TST to IGRA Testing in Ohio Prisons. [http://centerfortuberculosis.mayo.edu/uploads/7/1/7/3/71735537/_weiss_corrections.potx.pdf]. Accessed 7 Oct 2016.

[20] Vinton P, Mihrshahi S, Johnson P, Jenkin GA, Jolley D, Biggs BA (2009). Comparison of QuantiFERON-TB Gold in-tube test and tuberculin skin test for identification of latent mycobacterium tuberculosis infection in healthcare staff and association between positive test results and known risk factors for infection. Infect Control Hosp Epidemiol.

[21] Weinfurter P, Blumberg HM, Goldbaum G, Royce R, Pang J, Tapia J, Bethel J, Mazurek GH, Toney S, Albalak R (2011). Predictors of discordant tuberculin skin test and QuantiFERON(R)-TB Gold In-Tube results in various high-risk groups. Int. J. Tuberc. Lung Dis.

[22] Pai M, Zwerling A, Menzies D (2008). Systematic review: T-cell-based assays for the diagnosis of latent tuberculosis infection: an update. Ann Intern Med.

[23] Stephan C, Wolf T, Goetsch U, Bellinger O, Nisius G, Oremek G, Rakus Z, Gottschalk R, Stark S, Brodt HR (2008). Comparing QuantiFERON-tuberculosis gold, T-SPOT tuberculosis and tuberculin skin test in HIV-infected individuals from a low prevalence tuberculosis country. AIDS (London, England).

[24] Jones S, de Gijsel D, Wallach FR, Gurtman AC, Shi Q, Sacks H (2007). Utility of QuantiFERON-TB Gold in-tube testing for latent TB infection in HIV-infected individuals. Int. J. Tuberc. Lung Dis.

[25] Guidelines for the prevention and treatment of opportunistic infections in HIV-infected adults and adolescents: *Mycobacterium tuberculosis* Infection and Disease. In.; 2013.

[26] Nienhaus A, Schablon A, Costa JT, Diel R (2011). Systematic review of cost and cost-effectiveness of different TB-screening strategies. BMC Health Serv Res.

[27] Shah M, DiPietro D, Greenbaum A, Ketemepi S, Martins-Evora M, Marsiglia V, Dorman SE (2012). Programmatic impact of QuantiFERON-TB Gold In-Tube implementation on latent tuberculosis diagnosis and treatment in a public health clinic. PLoS One.

[28] Kowada A, Takasaki J, Kobayashi N (2015). Cost-effectiveness of interferon-gamma release assay for systematic tuberculosis screening of healthcare workers in low-incidence countries. J. Hosp. Infect.

[29] Person AK, Goswami ND, Bissette DJ, Turner DS, Baker AV, Gadkowski LB, Naggie S, Erlandson K, Chen L, Lalani T (2010). Pairing QuantiFERON gold in-tube with opt-out HIV testing in a tuberculosis contact investigation in the Southeastern United States. AIDS Patient Care STDs.

