# Supplementary material for: Cost analysis of tuberculin skin test and the QuantiFERON-TB Gold In-tube test for tuberculosis screening in a correctional setting in Dallas, Texas, USA
Source: BMC Infect Dis. 2016 Oct 12;16:564. doi: 10.1186/s12879-016-1901-8 (PMC5062880; doi:10.1186/s12879-016-1901-8)
Supplement: Additional file 1: Table S1. — Decision tree probabilities and cost-analysis for LTBI screening at DCJ. This table includes all of the inputs utilized for the decision tree, including the probabilities and costs for each decision node. (DOCX 19 kb) [file 12879_2016_1901_MOESM1_ESM.docx]

Table S1. Decision Tree Probabilities and Costs for LTBI Screening at DCJ

| Variables | Probability, % | Costs, $ | Source |
| --- | --- | --- | --- |
| TST placement | 94.3 | 11.18 | Retail cost + Time in motion data |
| TST not placed (prior positive) | 5.7 | 0 | DCJ data |
| TST read | 72 | 7.68 | Calculated, Time in motion data |
| TST not read | 28 | 0 | Calculated |
| TST positive | 2.6 | 0 | Calculated |
| TST negative | 97.4 | 0 | Calculated |
| CXR done in TST positive | 80 | 31.11 | Calculated, Time in motion data |
| CXR not done in TST positive | 20 | 0 | Calculated |
| Released, returned within 12 mos | 22 | 0 | DCJ release data |
| Released, no return | 78 | 0 | DCJ release data |
| QFT-GIT drawn | 95 | 39.73 | Retail cost + Time in motion data |
| QFT-GIT not drawn | 5 | 0 | Calculated |
| QFT-GIT positive | 13 | 0 | Calculated |
| QFT-GIT negative | 85 | 0 | Calculated |
| QFT-GIT indeterminate | 2 | 0 | Calculated |
| Repeat QFT-GIT positive | 10 | 39.73 | Calculated |
| Repeat QFT-GIT negative | 10 | 39.73 | Calculated |
| CXR done in QFT-GIT positive | 72 | 31.11 | Calculated, Time in motion data |
| CXR not done QFT-GIT positive | 28 | 0 | Calculated |
| Released, no repeat test | 80 | 0 | Calculated |

(LTBI = latent TB infection; DCJ= Dallas County Jail; TST = tuberculin skin test; CXR= chest x-ray; QFT-GIT = QuantiFERON Gold In-tube, “Calculated” refers to calculations made using primary study data)
